# Substance P enhances the local activation of NK_1_R-expressing c-kit^+^ cardiac progenitor cells in right atrium of ischemia/reperfusion-injured heart

**DOI:** 10.1186/s12860-020-00286-x

**Published:** 2020-06-09

**Authors:** Yun-Mi Jeong, Xian Wu Cheng, Kyung Hye Lee, Sora Lee, Haneul Cho, Weon Kim

**Affiliations:** 1 Division of Cardiology, Department of Internal Medicine, Kyung Hee University Hospital, Kyung Hee University, Hoegi-dong, Dongdaemun-gu, Seoul, 130-701 Republic of Korea; 2 Department of Mechanical Engineering, Korea Polytechnic University, 237 Sangidaehak Street, Si-heung City, Republic of Korea; 3 The Department of Cardiology, Yanbian University Hospital, Yanji, China

**Keywords:** Substance P, Ischemia-reperfusion, C-kit^+^ cardiac progenitor cells, Neurokinin 1 receptor, right atrium

## Abstract

**Background:**

Localization of neurokinin 1 receptor (NK_1_R), the endogenous receptor for neuropeptide substance P (SP), has already been described for the right atrium (RA) of the heart. However, the biological role of SP/NK_1_R signal pathways in the RA remains unclear.

Sprague-Dawley rats were randomly divided into 4 groups (*n* = 22 each); subjected to sham, ischemia/reperfusion-injury (I/R), I/R with 5 nmole/kg SP injection (SP + I/R), and SP + I/R with 1 mg/kg RP67580 injection (RP, a selective non-peptide tachykinin NK_1_R antagonist) (RP/SP + I/R). The left anterior descending coronary artery was occluded for 40 min followed by 1 day reperfusion with SP or SP + RP or without either. After 1 day, both atria and ventricles as well as the heart apexes were collected.

**Results:**

SP promoted the expression of c-Kit, GATA4, Oct4, Nanog, and Sox2 in only the RA of the SP + I/R rats via NK_1_R activation. In agreement with these observations, NK_1_R-expressing c-Kit^+^ Nkx2.5^+^GATA4^+^ cardiac progenitor cells (CPCs) in the ex vivo RA explant outgrowth assay markedly migrated out from RA^1 day SP + I/R^ approximately 2-fold increase more than RA^1 day I/R^. Treatment of SP promoted proliferation, migration, cardiosphere formation, and potential to differentiate into cardiomyocytes. Using RP inhibitor, NK_1_R antagonist not only inhibited cell proliferation and migration but also reduced the formation of cardiosphere and differentiation of c-Kit^+^ CPCs.

**Conclusion:**

SP/NK_1_R might play a role as a key mediator involved in the cellular response to c-Kit^+^ CPC expansion in RA of the heart within 24 h after I/R.

## Background

In 2003, the existence of resident cardiac progenitor cells (CPCs), which express c-Kit, GATA4, and Isl-1 and do not express CD45, CD31, CD34, or tryptase, were identified in adult heart tissue [1–3]. The resident c-Kit^+^ CPCs are known to be present in the atria, apex, and ventricle [1–3]. Most resident c-Kit^+^ CPCs are quiescent in the healthy adult myocardium [2]. The number of CPCs increases in response to cardiac repair following injury [2]. The frequency of CPCs is generally higher in the right atrium (RA) than in the left atrium (LA) and left ventricle (LV) [3]. A previous study compared the functional characteristics of RA CPCs, endothelial progenitor cells, and LV CPCs derived from the same patients (*n* = 14) [4]. RA CPCs showed greater expansion and migration potential compared to LV CPCs within the same patients [4]. Another study has demonstrated that RA CPCs from autologous right atrial appendages induce cardiac repair and blood vessel regeneration, raising high hopes for the treatment of ischemic heart disease via successful CPC engraftment and cardiomyogenic differentiation [5].

The RA is a chamber of the heart that receives all of the dark-red deoxygenated blood returning from circulation and then delivers it to the right ventricle (RV) [6, 7]. Chest radiography, echocardiography, computed tomography, and magnetic resonance imaging provide valuable atrial depictions that aid in the evaluation of the RA. These imaging technologies along with observation of associated clinical symptoms of RA are key to deflecting false-positive diagnosis and detecting missed findings [6, 7]. However, these tools alone are not able to reveal the underlying molecular mechanisms at work in the development and treatment of a damaged heart. Several studies have suggested that right atrial engineered heart tissue, spurred by corresponding growth factors and receptor systems, could help repair an infarcted heart, though prior research has not specifically investigated the role that local activation of RA c-Kit^+^ CPCs may play in healing a damaged heart [7, 8].

Neuropeptide substance P (SP) and its neurokinin-1 receptor (NK_1_R) are considered to have cardioprotective benefits for cardiac repair [9–11]. The presence of NK_1_R is known to be higher in the atria than in the ventricles [12]. However, the functions of SP/NK_1_R under ischemia/reperfusion injury (I/R) in the atria are not well understood. Previous studies have identified the priming effect of SP on different stem cell types, including neural stem cells (SCs), epithermal SCs, bone marrow-derived mesenchymal SCs (BMSCs), adipose derived stem cells (ADSCs), pluripotent tendon cells, and c-Kit^+^ cells [11, 13–16]. Despite the observed positive effects of SP on stem cell activation, the mechanism of SP/NK_1_R signaling in resident CPCs for cardiac repair remains unclear. The aim of this study was to explore whether SP/NK_1_R signaling triggers the awakening of quiescent resident CPCs in the heart after I/R. The present study was performed to characterize the expansion of resident CPCs by SP in each heart chamber within 24 h after I/R.

## Results

### SP enhances expansion of resident c-kit^+^ CPC in RA of I/R-injured heart through NK_1_R and pluripotency gene expression

**Fig. 1:**
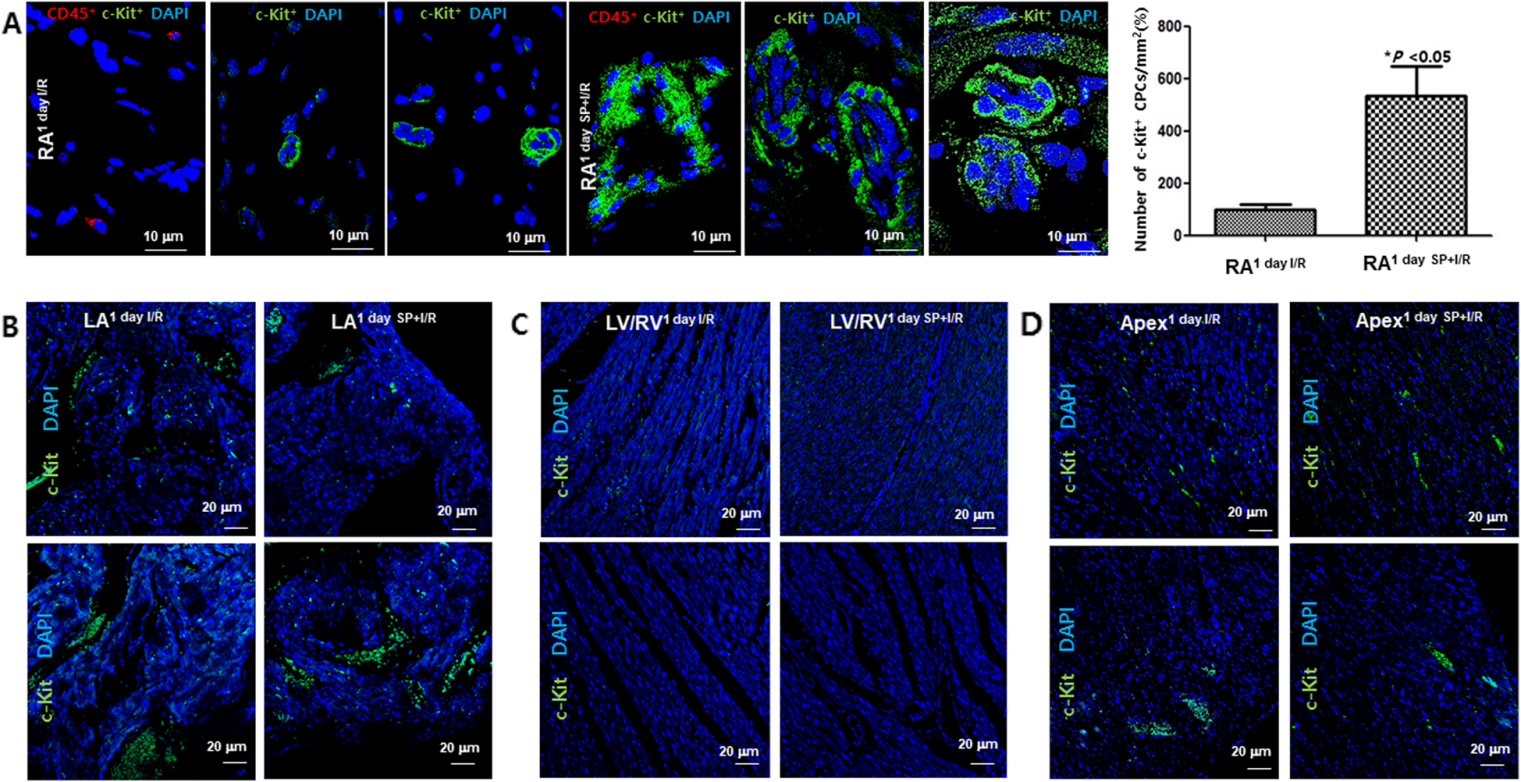
SP induces enrichment of CD45^−^c-Kit^+^ CPCs in the RA^1 day SP + I/R^ via high expression of c-Kit. (A) Confocal microscopy images showing c-Kit (green)-expressing CPCs in the RA^1day I/R^ and RA^1day SP + I/R^ groups. c-Kit^+^ CPCs (green), CD45^+^ cells (Red), and DAPI nuclei (blue) are visualized. Scale bars, 10 μm. Images were taken with a 100x oil immersion objective using an inverted Zeiss Axio Observer Z1 microscope. Graph showing the percentage of the number of cells per mm^2^ of c-Kit^+^ CPCs in the RA^1 day I/R^ and RA^1 day SP + I/R^ expressed as a percentage difference compared to the corresponding control. *P < 0.05 versus corresponding control using Student’s *t*-test. (B) Confocal microscopy images showing c-Kit (green)-expressing CPCs in the LA^1day I/R^ and LA^1day SP + I/R^ groups. (C) Confocal microscopy images showing c-Kit (green)-expressing CPCs in the LV/RV^1day I/R^ and LV/RV^1day SP + I/R^ groups. (D) Confocal microscopy images showing c-Kit (green)-expressing CPCs in the Apex^1day I/R^ and Apex^1day SP + I/R^ groups. Scale bars, 20 μm

**Fig. 2:**
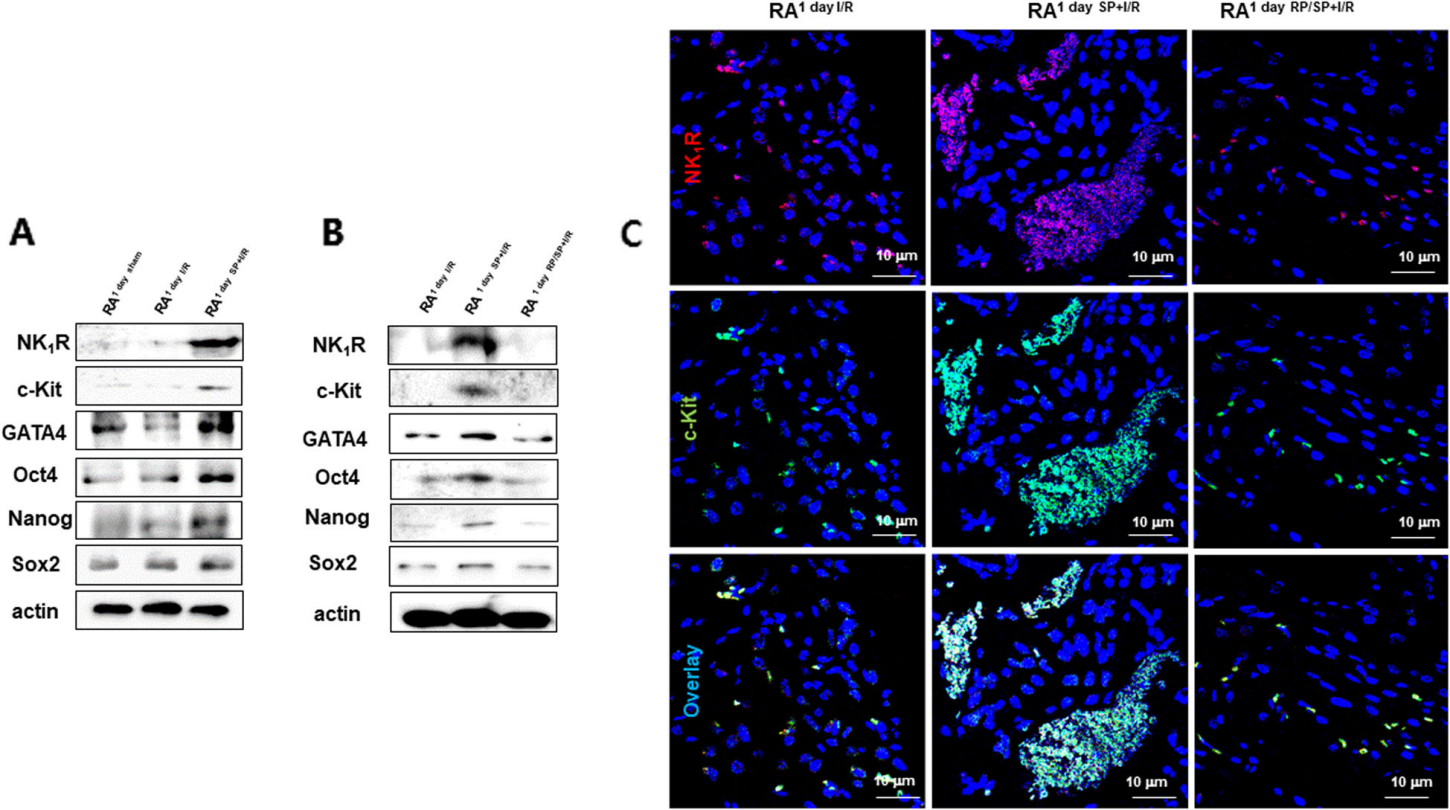
SP/NK_1_R signaling reveals cooperativity of c-Kit, GATA4, Oct4, Nanog, and Sox2 protein in the RA^1 day SP + I/R^. (A and B) Western blot analysis of c-Kit, GATA4, Oct4, Nanog, Sox2, and NK_1_R for RA^1 day sham^, RA^1 day I/R^, RA^1 day SP + I/R^ and RA^1 day RP/SP + I/R^. Actin was used as a loading control. (C) IFS analysis showing the expression of NK_1_R (red) and c-Kit (green) in RA^1 day I/R^, RA^1 day SP + I/R^, and RA^1 day RP/SP + I/R^. Scale bars, 10 μm

**Fig. 3:**
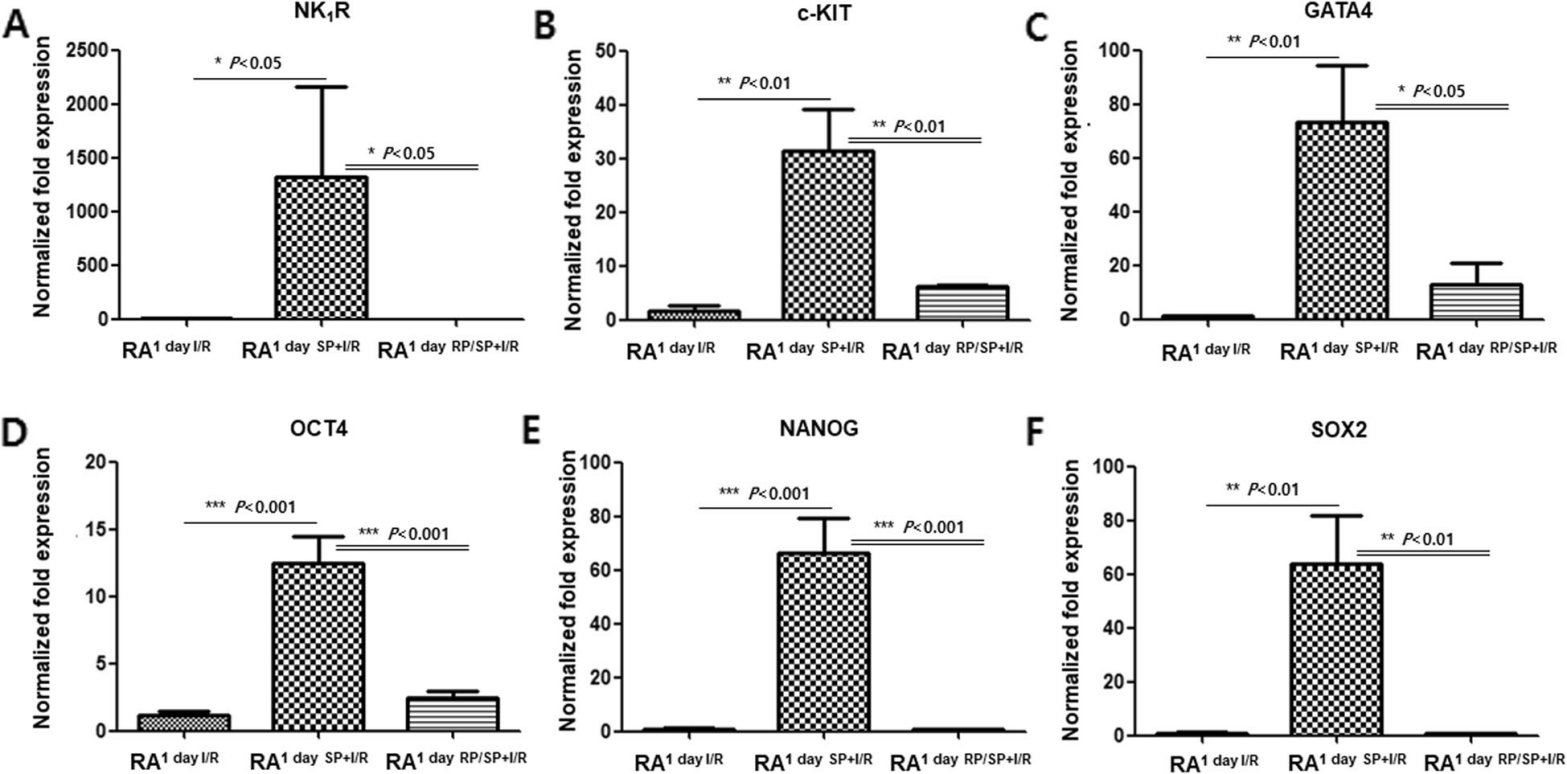
Inactivation of NK_1_R negatively correlates with expression of cardiac and pluripotency marker genes. (A-F) qRT-PCR bar graphs to quantify the expression of the indicated genes in RA^1 day I/R^, RA^1 day SP + I/R^, and RA^1 day RP/SP + I/R^. Data was analyzed using one-way analysis of variance (ANOVA) followed by Tukey’s post hoc *tests*. *P < 0.05, **P < 0.01, and ***P < 0.001 versus corresponding controls

Echo showed that exogenous SP significantly reduced infarct size and improved LV functional recovery in an I/R-injured heart (Fig. S1A), which is consistent with previous studies [9, 10]. SP significantly improved cardiac function of I/R rats that blunted the reductions of EF and FS caused by I/R injury (*P < 0.001* vs. I/R group). TTC staining demonstrated that the infarct size of SP + I/R hearts at 7 days significantly decreased by 20% (Fig. S1B). To explore whether SP/NK_1_R signaling is involved in the cellular response of resident c-Kit^+^ CPC to extensive cardiac repair, we screened reliable cardiac or stem cell marker genes on both atria and ventricles as well as the apexes of I/R and SP + I/R rats at 1 day using qRT-PCR assays. Upregulations of c-KIT, GATA4, NANOG, SOX2, and OCT4 mRNA were found in the RAs of SP + I/R-injured hearts, but not in the other heart chambers (Fig. S1C and D), suggesting that exogenous SP stimulates resident CPC accumulation into the RA of I/R-injured hearts. RA is known to be a good source of c-Kit^+^ CPC [3, 4]. We examined whether the up-regulation of stem cell markers in RA^1 day SP + I/R^ could be due to the increased c-Kit^+^ CPC population. Representative confocal images showed enrichment of c-Kit^+^ CPC/CD45^−^ in RA^1 day SP + I/R^, indicating a high level of c-Kit expression (Fig. 1A). Other chambers revealed no difference in c-Kit^+^ CPC population changes between the I/R and SP + I/R groups (Fig. 1B–D). Western blot analysis further confirmed that expression of c-Kit, GATA4, Nanog, Sox2, Oct4 as well as NK_1_R was more elevated in the RA^1 day SP + I/R^ than in the RA^1 day I/R^ (Fig. 2A). We next used RP to further verify whether the expansion of resident RA c-Kit^+^ CPC was at least in part due to SP/NK_1_R signaling. At the mRNA and protein levels, RP prevented these changes, with the RA^1 day SP + I/R^ exhibiting down-regulation of SP/NK_1_R-associated with c-Kit, GATA4, Nanog, Sox2, and Oct4 (Fig. 2B, C, and Fig. 3). These results provide evidence that SP might accelerate the expansion of resident c-Kit^+^ CPCs in the RA after I/R, collaborating with NK_1_R signaling and pluripotency gene expression.

### SP boosts cell proliferation, migration, the formation of cardiosphere, and differentiation of c-kit^+^ CPCs

**Fig. 4:**
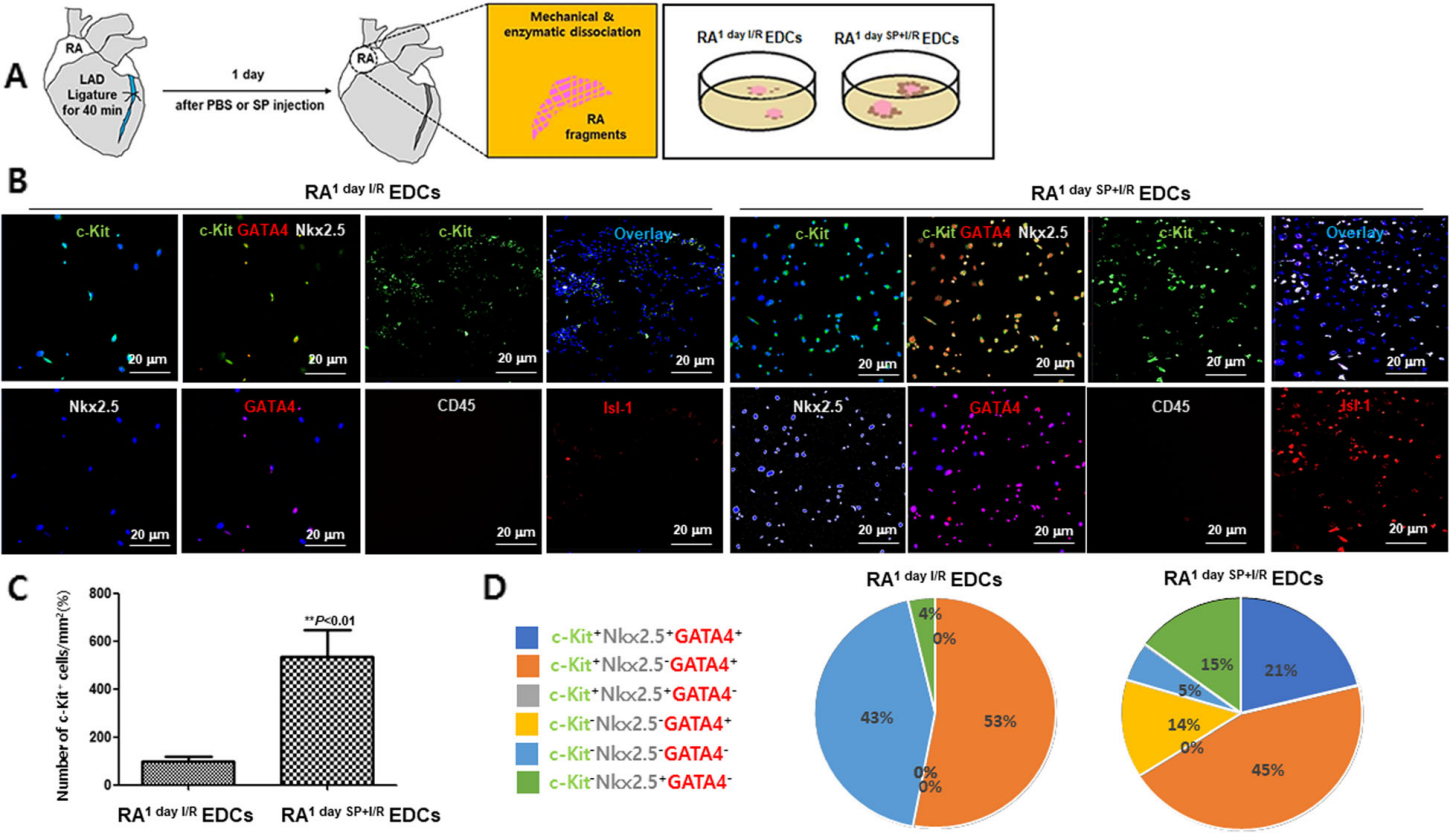
RA^1 day SP + I/R^ EDCs show an increased number of c-Kit^+^ GATA^+^ Nkx2.5^+^ CPCs. (A) Schematic workflow of the protocol for RA^1 day I/R^ and RA^1 day SP + I/R^ EDCs using ex vivo explant outgrowth culture assay as described in the Materials and Methods. (B) Confocal fluorescence images of NK_1_R-expressing c-Kit^+^ CPCs in each group. c-Kit (green), GATA4 (red), Nkx2.5 (gray), CD45 (gray), Isl-1 (red), and Hoechst33342 nuclei (blue) are visualized. Scale bars, 20 μm. (C) Graph depicting the percentages of c-Kit^+^ CPCs in RA^1 day I/R^ and RA^1 day SP + I/R^ EDCs. **P < 0.01 versus corresponding control using Student’s *t*-test. (D) Diagram indicating the percentages of c-Kit^+^Nkx2.5^+^GATA4^+^(), c-Kit^+^Nkx2.5^−^GATA4^+^(), c-Kit^+^Nkx2.5^+^GATA4^−^(), c-Kit^−^Nkx2.5^−^GATA4^+^(), c-Kit^−^Nkx2.5^+^GATA4^−^(), c-Kit^−^Nkx2.5^−^GATA4^−^() in RA^1 day I/R^ and RA^1 day SP + I/R^ EDCs.

**Fig. 5:**
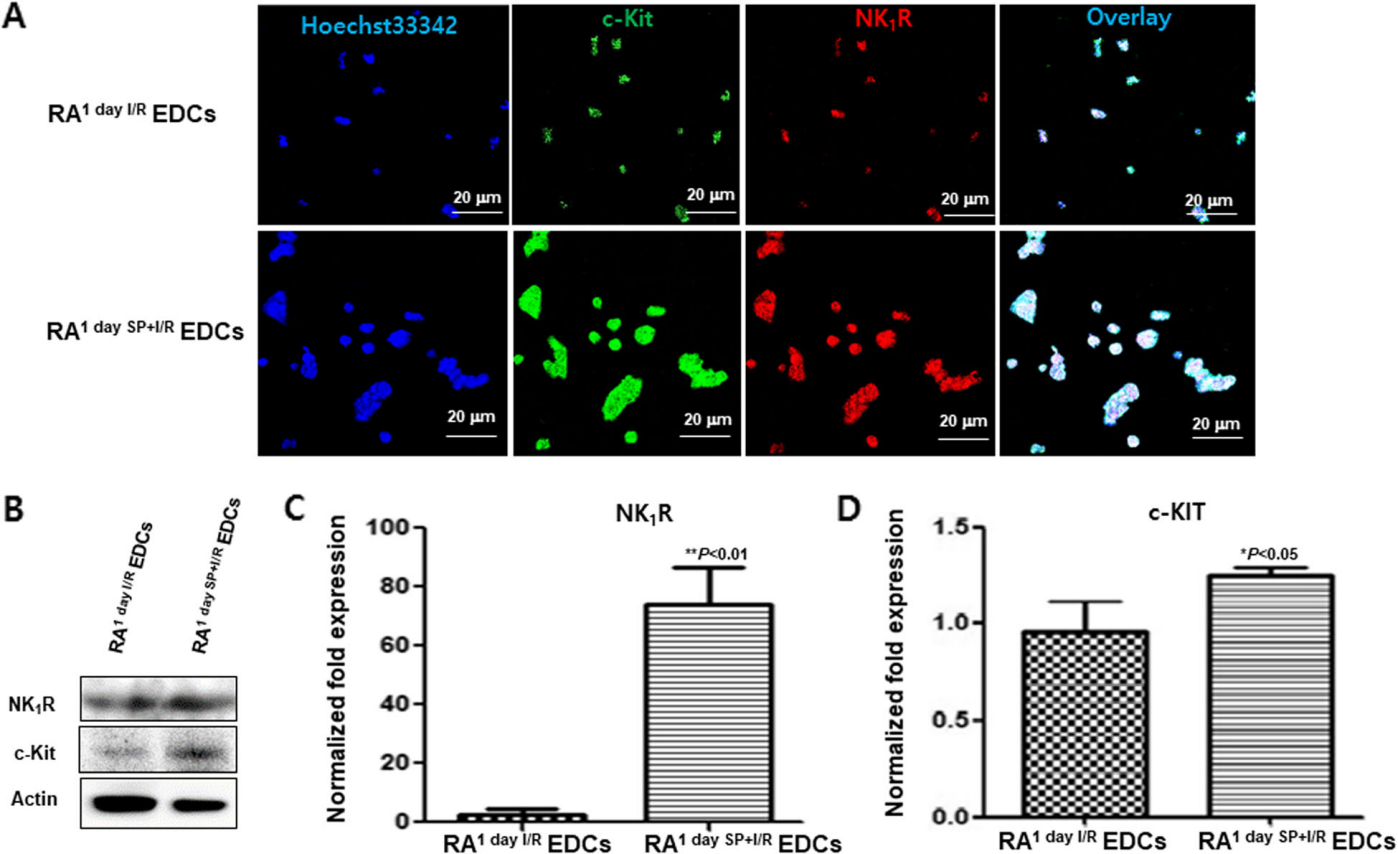
Co-expression of c-Kit and NK_1_R in RA^1 day I/R^ and RA^1 day SP + I/R^ EDCs. (A) Confocal fluorescence images of NK_1_R-expressing c-Kit^+^ CPCs in RA^1 day 1/R^ EDCs and RA^1 day SP + 1/R^ EDCs. c-Kit (green), NK_1_R (Red), Hoechst33342 nuclei (blue) are visualized. Scale bars, 20 μm. (B) Western blot analysis to detect the expression of proteins NK_1_R and c-Kit in each group. Actin was used as a loading control. (C and D) qRT-PCR analysis graphs showing the mRNA expression of NK_1_R and c-Kit in RA^1 day 1/R^ EDCs and RA^1 day SP + 1/R^ EDCs. The bars represent the mean ± SD of triplicate assays expressed as percentages of the RA^1 day I/R^ EDCs. *P < 0.05 and **P < 0.01 versus corresponding controls

If SP/NK_1_R signaling switches on the expression of c-Kit, GATA4, and pluripotency genes, resident RA c-Kit^+^ CPCs might expand to more than double its population by SP. To test this, the RA^1 day I/R^ and RA^1 day SP + I/R^ fragments were cultured by ex vivo explant outgrowth assay for 2 weeks, as shown in Fig. 4A. Expended c-Kit^+^ CPCs derived from RA^1 day I/R^ and RA^1 day SP + I/R^ fragments were evaluated using confocal microscopy with c-Kit, GATA4, NKx2.5, Isl-1, CD45, and cTnl antibodies. Explant derived cells (EDCs) of RA^1 day SP + I/R^ showed notably high expression of c-Kit and Isl-1 (Fig. 4B and C). As shown in Fig. 4B and D, the percentage of c-Kit^+^GATA4^+^NKX2.5^−^ or c-Kit^−^GATA4^−^NKX2.5^−^ cells in RA^1 day I/R^ were higher than RA^1 day SP + I/R^ EDCs, whereas the percentage of c-Kit^+^GATA4^+^NKX2.5^+^, c-Kit^−^GATA4^+^NKX2.5^−^, c-Kit^−^GATA4^−^NKX2.5^+^, and c-Kit^−^GATA4^−^NKX2.5^−^ cells was lower than 5% (Fig. 4D). Of note, the percentage of c-Kit^+^GATA4^+^NKX2.5^+^, c-Kit^−^GATA4^+^NKX2.5^−^, c-Kit^−^GATA4^−^NKX2.5^+^ cells in RA^1 day SP + I/R^ EDCs was increased (Fig. 4B-D), which the expression of CD45 and troponin I did not (Fig. 4B and Fig. S2). Furthermore, IFS, western blot analysis, and qRT-PCR showed increased levels of c-Kit and NK_1_R expression in the RA^1 day SP + I/R^ EDCs (Fig. 5). Interestingly, the SP-treated EDCs appeared to have much larger nuclear than EDCs alone (Fig. 5A). These results suggest that SP may play an important key in stimulating the active state of resident c-Kit^+^ CPCs.

**Fig. 6:**
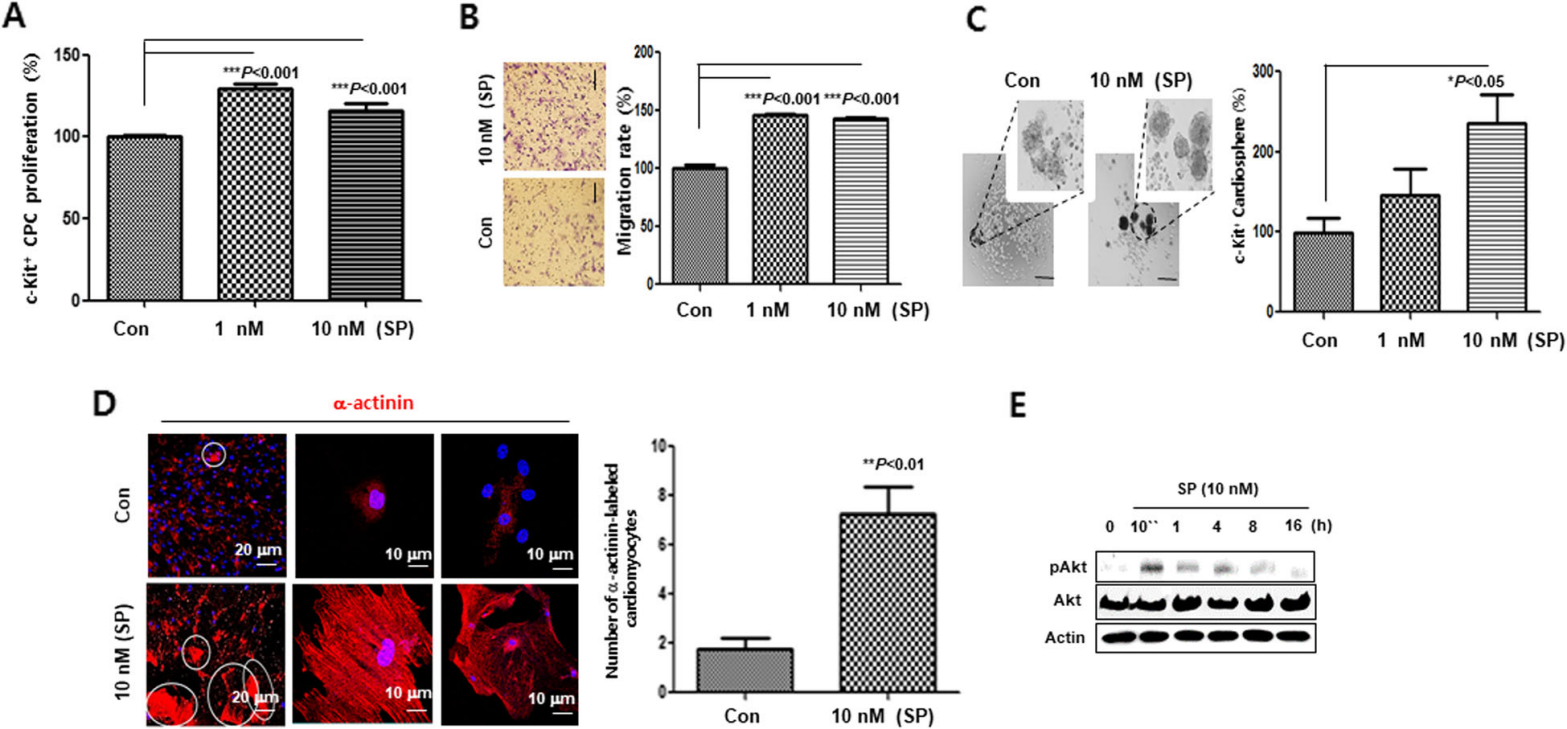
SP boosts cardiogenic potential of RA c-Kit^+^ CPCs. (A) c-Kit^+^ CPC proliferation in the presence or absence of SP (1 and 10 nM) was measured using the EZ-Cytox cell viability assay kit. ***P < 0.001, versus control using a one-way analysis of variance (ANOVA) followed by Tukey’s post hoc *tests*. (B) Phase contrast images of transwell migration assay. Scale bars, 20 μm. Graph indicating the rate of cell migration. *** P < 0.001, versus control using a one-way analysis of variance (ANOVA) followed by Tukey’s post hoc *tests*. (C) Images of morphology of cardiosphere from purified c-Kit^+^ CPCs with or without SP. Graphs showing the number of cardiospheres for the control, 1 nM, and 10 nM of SP. *P < 0.05, versus control using a one-way analysis of variance (ANOVA) followed by Tukey’s post hoc *tests*. (D) Confocal images of α-actinin-labeled cardiomyocytes (red). Scale bars 20 μm and 10 μm. Graphs representing the total number of α-actinin-labeled cardiomyocytes in control vs SP (10 nM)-treated c-Kit^+^ CPCs at 4 weeks. **P < 0.01 versus control using Student’s *t*-test. (E) Western blot analysis showing the activation of Akt and total Akt in SP (10 nM)-treated c-Kit^+^ CPCs at the indicated time points

To better understand the effect of SP on c-Kit^+^ CPCs activation, we next purified c-Kit^+^ CPCs from RA EDCs by MACS methods using anti-c-Kit antibody and characterized them (Fig. S3). The cells were treated with SP in order to evaluate the effects of SP on the proliferation, migration, cardiosphere formation, and cardiomyocyte differentiation properties of NK_1_R-expressing c-Kit^+^ CPCs. In the presence of SP, NK_1_R-expressing c-Kit^+^ CPCs exhibited an approximately 20% increase in cell proliferation (Fig. 6A). SP-treated NK_1_R-expressing c-Kit^+^ CPCs also exhibited significantly higher migration rates than that of the control (Fig. 6B). Furthermore, SP led to a significant increase in cardiosphere formation (Fig. 6C). SP-treated c-Kit^+^ CPCs were more highly committed to cardiomyocyte differentiation, compared to the untreated control (Fig. 6). NK_1_R inhibitors prevented the proliferation, migration, cardiosphere formation, and cardiomyocyte differentiation of c-Kit^+^ CPC increased by SP (Fig. S4 and S5). If SP/NK_1_R accelerated these properties of c-Kit^+^ CPCs, it may also affect the expression of Akt, which is a key modulator in the expansion of the CPC population [17]. The phosphorylation of Akt was markedly activated 10 min after SP treatment (Fig. 6E). Taken together, these findings indicate that there is an association between SP/NK_1_R pathways and the activation of c-Kit^+^ CPC.

## Discussion

In the present study, our findings highlight the significant role of SP on local activation of NK_1_R-expressing c-Kit^+^ CPCs in the I/R-injured RA at 1 day. We found that NK_1_R is expressed in the nucleus of resident c-Kit^+^ CPCs in RA. SP treatment appeared to have affected the nucleus of EDCs. Although the specific roles of SP/NK_1_R in changing the ploidy or nuclear content of resident c-Kit^+^ CPCs in RA have not yet been validated, a previous study has proposed a possible link between SP/NK_1_R and nuclear delivery that are actively expressed in the target cells [18]. In addition, several studies have demonstrated the expression and/or properties of NK_1_R in various in vitro stem cells [12–15, 19–22]. For example, NK_1_R is highly expressed in the nucleus of ADSC [20]. After SP treatment, ADSC proliferation and mitogenesis increase through NK_1_R [20]. Similarly, SP significantly stimulates the proliferation of BMSC [15, 21]. The effects of SP on the proliferation of BMSC is inhibited by NK_1_R antagonists [21]. Another paper has demonstrated that cultured NK_1_R-expressing neural progenitor cells proliferate more under normal and ischemic conditions in the presence of SP [22]. Based on these previous studies, SP/NK_1_R might have a central role in the local activation of resident RA c-Kit^+^ CPCs after I/R-injury.

We demonstrated that SP promoted the stem cell properties of NK_1_R-expressing c-Kit^+^GATA4^+^Nkx2.5^+^/CD45^−^cTnI^−^ CPCs, which in turn was associated with high expression of pluripotency genes. It is well known that c-Kit and GATA4 expression is necessary and sufficient for cardiac recovery [2]. In the case of c-Kit-activated transgenic mice, activated c-Kit receptors can improve cardiac recovery after cryoinjury [2]. CD45^−^c-Kit^+^ isolated/cultured cardiac stem cells (CSCs) derived from the c-Kit-activated transgenic mice promote CSC activation and differentiation in vitro through Akt pathways [2]. Furthermore, c-Kit^+^ cardiac outgrowth cells transplanted into the damaged heart improve the LVEF and increase neovascularization [23]. However, in a recent study GATA4 and GATA6 were deleted from c-Kit^+^ CPCs to block all de novo cardiomyocyte formation [24]. Unexpectedly, the total number of cardiomyocytes increased in transgenic mice with the deletion of GATA4/6 or GATA4 [24]. Although several papers have demonstrated that GATA4 is able to potentiate gene expression programs associated with multiple cardiovascular lineages in priming c-Kit^+^ CPCs, this previous study demonstrated that GATA4-deleted adult cardiac endothelial cells increase progenitor differentiation toward an endothelial lineage [24].

Our results indicate how c-Kit expression by SP/NK_1_R can contribute to CPC activation by stimulating several transcription factors and signal pathways. Moreover, our study found evidence that SP promotes the proliferation, migration, cardiosphere formation, and differentiation of c-Kit^+^ CPCs derived from RA after I/R. This evidence is not enough to decisively demonstrate that SP is involved in the recovery of I/R-damaged LV. The present study has several limitations. For example, to detect RA c-Kit^+^ CPCs accumulated at least in the LV which had infarct zone, fluorescent dye-labelled cell trafficking tools should be developed in vivo model in long-term and real-time condition. The present study is not proper for c-Kit^+^ CPC tracking. Further research could investigate how SP activated NK_1_R-expressing c-Kit^+^ CPCs determine their routes to the damaged LV.

Another limitation of our study is that it only indirectly tracked the effect of SP on the local activation of RA c-Kit^+^ CPCs and their behavior in cardiac repair. There remain unresolved questions about the biological significance of c-Kit and GATA4 in the CPC-mended broken heart after myocardial infarction. Moreover, it remains controversial whether all c-Kit^+^ CPCs originate from bone marrow [25]. Despite the limitations of this study, additional studies based on SP-mediated stem cell activation and angiogenesis during ischemia in mice and in patients with acute myocardial infarction should be able to provide greater clarity [26]. Therefore, the effect of SP-activated NK_1_R-expressing c-Kit^+^ CPCs on RA of I/R-injury heart in the beginning should be further investigated to confirm the precise mechanisms of SP in the process from I/R injury to cardiac repair.

## Conclusions

Our findings offer a possible mechanism, the work of SP/NK_1_R on local activation of RA c-Kit^+^ CPC following I/R, and may provide insight on how SP contributes to initial response to cardiac healing.

## Methods

### Materials

SP (6.7 μg/kg/0.1 ml) was purchased from Sigma (St. Louis, MO, USA). RP67580 (RP, a selective non-peptide tachykinin NK_1_R antagonist, 1 mg/kg) was obtained from R&D Systems Inc. (Minneapolis, MN, USA). Hoechst33342 and DAPI were purchased from Thermo Fisher Scientific (Rockford, IL, USA). AccuPower®RocketScript™ Cycle RT PreMix (dN12) and AccuPower®ProFi Taq PCR PreMix were purchased from Bioneer (DaeJeon, Korea). SYBR®Green Mix was obtained from Applied Biosystems (Lincoln, CA, USA). Antibodies that recognize GATA4 (ab86371), Nkx2.5 (ab91196), Nanog (ab106465), or goat anti-rabbit IgG H&L (Alexa Fluor®488, 594, and 647) were purchased from Abcam (Cambridge, UK). Antibodies specific for c-Kit (sc5535), Oct-3/− 4 (sc5279), Sox2 (sc2008), Isl-1 (sc101072), NK_1_R (sc15323), CD45 (sc25590), or actin (sc47778) were obtained from Santa Cruz Biotechnology, Inc. (CA, USA). Antibodies specific for phosphor-Akt (Ser473) (#9271) and Akt (#9272) were purchased from Cell Signaling Technology (MA, USA).

### Animal experiment

Eight-week-old male Sprague-Dawley (SD) rats were used under a protocol approved by the Institutional Animal Care and Use Committee of Kyung Hee Medical Center (KHMC-IACUC:2015–028) [11, 27]. The SD rats were randomly divided into 4 groups (*n* = 22 each): sham, I/R, I/R with 5 nmole/kg SP injection (SP + I/R), and SP + I/R with 1 mg/kg RP injection (RP/SP + I/R). The SP and RP were injected via the tail intravenously as previously described [28, 29]. The left anterior descending coronary artery was occluded for 40 min followed by 1 day reperfusion with SP, with SP + RP, and without either. The SD rats were anaesthetized by using 2.5% isoflurane (Hana Pharm Co.,Ltd., Seoul, Korea). The rats were euthanized on 1 day, and the RA, LA, RV, LV, and apex derived from the heart samples were collected. According to Institutional Animal Care and Use Committee of Kyung Hee Medical Center standardized pain protocol, all SD rat continually was monitored for signs of distress. The SD rats were housed in the same pathogen-free facility under a 12 h light and dark cycle with ad libitum feeding. No more than three animals were housed per cage.

### RA ex vivo explant outgrowth culture assay

RA tissue were cut into 1 to 2 mm fragments, washed with Ca^2+^/Mg^2+^ − free PBS, and digested three times for 10 min with 0.2% trypsin and 0.1% collagenase at 37 °C [11, 30]. The suspended cells and RA fragments were incubated with complete medium [CM; Dulbecco’s modified Eagle’s medium supplemented with 10% ES cell grade FBS, 5% horse serum, 10 ng/ml LIF, 1% penicillin-streptomycin, fungizone, and gentamicin] at 37 °C in a 5% CO_2_ incubator. After 2 weeks, the attached cells, which were surrounding the explants having migrated out, were analyzed by immunofluorescence with anti-c-Kit antibody. The c-Kit^+^ CPCs were purified by a magnet-activated cell sorting system (MACS)(Dynal Biotech, Oslo, Norway) [30]. RA explant-derived cells (EDCs) were suspended in trypsin, incubated with anti-c-Kit antibody (1100), and separated using immunomagnetic microbeads (Dynal Biotech). c-Kit^+^ CPCs were cultured for 1 month with CM at 37 °C in a 5% CO_2_ incubator. The c-Kit^+^ CPCs of the P2 passages were used for all experiments.

### Cell proliferation assay

The cell proliferation assay was assessed using an EZ-Cytox cell viability assay kit (DoGEN, Seoul, Korea) [27]. After SP treatment for 7 days, the cultured medium was removed. Cells were stained with EZ-cytox solution for 1 h. Absorbance was determined at 490 nm using an ELISA reader (Emax; Molecular Devices, Sunnyvale, CA, USA).

### Cell migration assay

To determine the priming effects of SP on c-Kit^+^ CPC migration, a cell migration assay was performed using 0.8 μm pore size, and 24 well transwell migration chambers coated with Type IV collagen (10 μg/ml) as previously described [27]. 1 × 10^4^ c-Kit^+^ CPCs were seeded into the upper transwell chambers containing medium. Then the chamber was inserted into each well of 24-well plates containing 600 μl medium supplemented with or without SP at the indicated concentration. The chambers were then incubated for 24 h at 37 °C in a 5% CO_2_ incubator. The cells that migrated to the outer side of the membrane were stained with a crystal violet staining solution. The absorbance was determined at 590 nm using an ELISA reader (Emax; Molecular Devices, Sunnyvale, CA, USA).

### Cardiosphere formation

c-Kit^+^ CPCs were incubated in Dulbecco’s MEM and Ham’s F12 (ratio 1:1; Sigma), bFGF (10 ng/ml), EGF (20 ng/ml), LIF (10 ng/ml), insulin-transferrin-selenite (Gibco), 1x B27 (Gibco), 1x N2 (Gibco), 1% penicillin-streptomycin, 1% fungizone, and gentamicin in the presence or absence of SP at the indicated concentration [30]. After 2 weeks, the cardiosphere formation of c-Kit^+^ CPCs was visualized with an Olympus BX51 microscope equipped with a 20x lens. The number of spheres was counted manually from brightfield images using the ImageJ cell counter plugin and expressed as a percentage. All bright-field images were selected with clone identities blinded and at least 20 random images were obtained from each well.

### Cardiomyocyte differentiation

The medium for cardiomyocyte differentiation was composed of MEM Alpha, 10% FBS supplemented with 1 μM dexamethasone (Sigma), and 1 mM β-glycerophosphate (Sigma). The c-Kit^+^ CPCs were incubated with or without SP (10 nM) in cardiomyocyte differentiation medium for 4 weeks. Cardiomyocyte differentiation was determined by immunofluorescence staining with anti-α-actinin (1:100) antibody [30].

### Quantitative reverse-transcription PCR (qRT-PCR)

cDNA was synthesized using AccuPower®RocketScript™ Cycle RT PreMix (dN12) (Bioneer, DaeJeon, Korea). qRT-PCR assays were carried out with SYBR®Green Mix and the appropriate primers (Applied Biosystems), and were run on a StepOnePlus real-time PCR system (Applied Biosystems). The relative gene expression from all data were obtained using the ΔCt method with normalization versus RPL-32 as previously described [27, 30]. The primers used were: NK_1_R (Forward-TACACTGTGGGGCCAGTGAGATC, Reverse-GGTACACACAACCACGATCATCA); c-KIT (Forward-AGACGTACAGATCCAGAATG, Reverse-TGCTCTTTGCTGTTACCTT); NANOG (Forward-CTCTCTACCATTCTGAACCTGAGC, Reverse-TCAGGCCGTTGCTAGTCTTC); OCT4 (Forward-GCCCCCATTTCACCACACT, Reverse-CCAGAGCAGTGACAGGAACA); SOX2 (Forward-GACAGCTACGCGCACATGAA, Reverse-CGAGCTGGTCATGGAGTTGT); GATA4 (Forward-ACCCTGCGAGACACCCCAAT, Reverse-GTAGAGGCCACAGGCGTTGC); RPL-32 (Forward- TGTCAAGGAGCTGGAAGTGC, Reverse-AGGCACACAAGCCATCTATTCA).

### Immunofluorescence staining (IFS)

The tissue samples were fixed with 4% paraformaldehyde, embedded with paraffin, and sectioned into 7 μm-thick sections. The c-Kit^+^ CPCs were fixed with 4% paraformaldehyde. They were stained with standard IFS methods as previous described [2, 27, 30]. After nuclear DAPI or Hoechst 33342 staining, immunostained confocal images were acquired using an inverted Zeiss Axio Observer Z1 microscope with 405, 458, 488, 514, 561, and 633 nm laser lines. To calculate the quantification of c-Kit^+^ CPCs, we used microscope software ZEN from ZEISS microscopy which can count the number of c-Kit^+^ CPCs and number of total cells per field.

### Western blot analysis

The frozen samples were disrupted using the TissueLyser II (Qiagen), after which an ice-cold PRP-PREP protein extraction solution with a protease inhibitor cocktail (iNtRON Biotechnology, Inc., Seoul, Korea) was added, and the samples were homogenized by stainless steel beads (Qiagen, Cam USA). Protein concentration was assessed using the BCA-kit (Thermo Scientific, Rockford, IL, USA). An equal amount of protein (80 μg) from each sample was loaded onto 10 to 12% SDS gel, and transferred to a PVDF membrane (Merk Millipore, MA, USA). The membranes were blocked for 2 h at room temperature with 5% nonfat dry milk in PBS containing 0.1% Tween-20, and incubated with primary antibodies (1:1000 and 1:500) overnight at 4 °C. After washing three times, the membranes were incubated with a horseradish peroxidase-conjugated secondary antibody (1:5000) at RT for 2 h and visualized with a chemiluminescence substrate.

### Statistical analysis

Student’s *t-*tests (for comparisons of two groups) or a one-way analysis of variance (ANOVA) (for comparisons of three or more groups) followed by Tukey post hoc tests were used for the statistical analyses. SPSS software ver. 17.0 (SPSS, Chicago, IL) was used. A value of *P < 0.05* was considered significant. Data are expressed as means ± standard error of the mean (SEM). Data analysis was carried out using the GraphPad Prism software (GraphPad Software Inc). *P < 0.05–0.01, **P < 0.01–0.001, and ***P < 0.001 vs. corresponding controls. All error bars represent the standard deviation of three or more biological replicates.

## Supplementary information

**Additional file 1: Fig. S1.** The effects of SP on I/R-injured heart. Fig. S2. RA^1 day I/R^ EDCs and RA^1 day SP + I/R^ EDCs did not show cTnl expression. **Fig. S3.** Characterization of NK_1_R-expressing c-Kit^+^ CPCs. **Fig. S4.** NK_1_R inhibitor blocks SP-stimulated the cell proliferation, migration, and cardiosphere formation of RA c-Kit^+^ CPCs. **Fig. S5**. NK_1_R inhibitor blocks SP-stimulated the cardiomyocyte differentiation of RA c-Kit^+^ CPCs.

## Data Availability

All results generated or analyzed during present study are included in this published article and its supplementary information files. Data and materials will be made available upon request via email to first author (phdjeongym12@kpu.ac.kr).

## Abbreviations

CPCs: Cardiac progenitor cells
RA: Right atrium
LA: Left atrium
LV: Left ventricle
RV: Right ventricle
SP: Substance P
NK1R: Neurokinin-1 receptor
I/R: Ischemia/reperfusion injury
SCs: Stem cells
BMSCs: Bone marrow-derived mesenchymal SCs
ADSCs: Adipose derived stem cells
EDCs: Explant-derived cells
IFS: Immunofluorescence staining
