# Supplementary material for: Substance P enhances the local activation of NK1R-expressing c-kit+ cardiac progenitor cells in right atrium of ischemia/reperfusion-injured heart
Source: BMC Mol Cell Biol. 2020 Jun 9;21:41. doi: 10.1186/s12860-020-00286-x (PMC7285458; doi:10.1186/s12860-020-00286-x)

**Substance P enhances the local activation of NK_1_R-expressing** **c-Kit^+^ cardiac progenitor cells in right atrium of ischemia/reperfusion-injured heart**

Yun-Mi Jeong^1,3^, Xian Wu Cheng^2^, Kyung Hye Lee^1^, Sora Lee^1^, Haneul Cho^1^, Weon Kim^1,*^

^1^Division of Cardiology, Department of Internal Medicine, Kyung Hee University Hospital, Kyung Hee University, Seoul, Republic of Korea

^2^ The Department of Cardiology, Yanbian University Hospital, Yanji, China

^3^ Department of Mechanical Engineering, Korea Polytechnic University, 237 Sangidaehak Street, Si-heung City, Republic of Korea

***Corresponding author:**

Weon Kim, MD, PhD

Department of Cardiovascular of Internal Medicine

Kyung Hee University Hospital, Kyung Hee University

Hoegi-dong, Dongdaemun-gu, Seoul, 130-701, Republic of Korea

Tel.: +82 2 958 8176

Fax: +82 2 958 8160

E-mail address: [mylovekw@hanmail.net](mailto:mylovekw@hanmail.net)

**Methods**

**Echocardiography (Echo)**

After 7 days of reperfusion of I/R and SP+I/R, a 15-MHz transducer with a commercially available system (Sonos 5500, Philips Ultrasound, Bothell, WA, USA) was used to measure the ejection fraction (EF), fractional shortening (FS), the LV mass index (LVMI), LV end-diastolic volume (LVEDV), LV end-systolic volume (LVESV), LV end-diastolic dimension (LVEDD), and LV end-systolic dimension (LVESD). All echo was performed by a cardiac echocardiographic specialist. After image acquisition, a cardiologist who specializes in echo analyzed the echocardiographic images.

**Measurement of infarct size**

Infarct sizes at 7 days after reperfusion were measured. Tissue samples were incubated for 20 min at 37°C with 1% 2,3,5-tripheyltetrazolium chloride (TTC) in 0.1 mol/L phosphate buffer (pH 7.4), and photographed with a digital camera. Red and pale unstained areas of every slice were also photographed with a digital camera, and the infarct area was quantified using an ImageJ software. Infarct size is expressed as a percentage of the LV area.

**c-Kit^+^ CPC proliferation assay**

The c-Kit^+^ CPC proliferation assay was assessed using an EZ-Cytox cell viability assay kit (DoGEN, Seoul, Korea)^16^. After SP or SP/RP treatment for 7 days, the cultured medium was removed. Cells were stained with EZ-cytox solution for 1 h. Absorbance was determined at 490 nm using an ELISA reader (Emax; Molecular Devices, Sunnyvale, CA, USA).

**c-Kit^+^ CPC migration assay**

To determine the priming effects of SP on c-Kit^+^ CPC migration, a cell migration assay was performed using 0.8 μm pore size, and 24 well transwell migration chambers coated with Type IV collagen (10 μg/ml) as previously described [16]. 1x10^4^ c-Kit^+^ CPCs were seeded into the upper transwell chambers containing medium. Then the chamber was inserted into each well of 24-well plates containing 600 μl medium supplemented with SP or SP/RP the indicated concentration. The chambers were then incubated for 24 h at 37°C in a 5% CO_2_ incubator. The cells that migrated to the outer side of the membrane were stained with a crystal violet staining solution. The absorbance was determined at 590 nm using an ELISA reader (Emax; Molecular Devices, Sunnyvale, CA, USA).

**Cardiosphere formation**

c-Kit^+^ CPCs were incubated in Dulbecco’s MEM and Ham’s F12 (ratio 1:1; Sigma), bFGF (10 ng/ml), EGF (20 ng/ml), LIF (10 ng/ml), insulin-transferrin-selenite (Gibco), 1x B27 (Gibco), 1x N2 (Gibco), 1% penicillin-streptomycin, 1% fungizone, and gentamicin in the presence or absence of SP or SP/RP. After 2 weeks, the formation of the cardiosphere was determined by counting the number of clones analysis with a c-Kit antibody [19].

**Cardiomyocyte differentiation**

The medium for cardiomyocyte differentiation was composed of MEM Alpha, 10% FBS supplemented with 1 μM dexamethasone (Sigma), and 1 mM β-glycerophosphate (Sigma). The c-Kit^+^ CPCs were incubated with or without SP (10 nM) in cardiomyocyte differentiation medium for 4 weeks. Cardiomyocyte differentiation was determined by immunofluorescence staining with anti-α-actinin (1:100) antibody [19].

**Quantitative reverse-transcription PCR (qRT-PCR)**

cDNA was synthesized using AccuPower®RocketScript^TM^ Cycle RT PreMix (dN12) (Bioneer, DaeJeon, Korea). qRT-PCR assays were carried out with SYBR®Green Mix and the appropriate primers (Applied Biosystems), and were run on a StepOnePlus real-time PCR system (Applied Biosystems). The relative gene expression from all data were obtained using the ΔCt method with normalization versus RPL-32. The primers of quantitative reverse-transcription PCR (qRT-PCR) used were:

|  | forward | reverse |
| --- | --- | --- |
| ISL-1 | AGTCCGGAGAGACATGATGG | TGCAAGGCGAAGTCACTCAG |
| c-KIT | TCCCGCCAGTAGACGTACAG | ACAGCGTTGGAGGCCTTACA |
| SCA1 | GCACGATGATCCCATTTGGT | GACGGGAAGGCAAATATGGT |
| OCT4 | CTGACAACAACGAGAACCTT | CTGCTTGGCAATGCTAGTGA |
| KIF4 | GATGGGGTCTGAGACTGGAT | AACTTCCAGTCACCCCTTGG |
| NANOG | CTCTCTACCATTCTGAACCTGAGC | TCAGGCCGTTGCTAGTCTTC |
| SOX2 | AACTCCATGACCAGCTCGCA | CTGGAGTGGGAGGAAGAGGT |
| ACTIN | CGCGGGTACTCCTTTGTCA | CCA GGG CGA CAT AAC ACA GT |
| GATA4 | ACCCTGCGAGACACCCCAAT | GTAGAGGCCACAGGCGTTGC |
| NKX2.5 | AGCGGCGCTTCAAGCAAC | ACCAGATCTTGACCTGCGTG |
| MEF2C | CGAGATACCCACAACACACG | GGAGTGGAATTCGTTCCGGT |
| NK-1R | TACACTGTGGGCCAGTGAGATC | GGTACACACAACCACGATCATCA |
| PECAM1 | CGCTGGTGTACAACGTCTCCT | GCTGTTCAGTATCACGGTGCAT |
| vWF | CACTTGCTCCTGTGAGTCCA | ACAGTGTGGGTTTCCTCCAG |
| SMA | GCTCCTCCAGAACGCAAATATT | CCAGCTTCGTCATACTCCTGT |

**Immunofluorescence staining (IFS) of RA^1 day I/R^ EDCs and RA^1 day SP+I/R^ EDCs**

The c-Kit^+^ CPCs were fixed with 4% paraformaldehyde. They were stained with standard IFS methods with c-TnI. After nuclear DAPI or Hoechst 33342 staining, immunostained confocal images were acquired using an inverted Zeiss Axio Observer Z1 microscope with 405, 458, 488, 514, 561, and 633 nm laser lines.

**legends**

**Figure S1. The effects of SP on I/R-injured heart.** (A) a time-line diagram for workflow of animal model. (B) Representative M-mode images of echo derived from sham, I/R, and SP+I/R at 7 days. N=6/each group. Data was analyzed using one-way analysis of variance (ANOVA) followed by Tukey’s *post hoc tests*. ****P<0.001* versus I/R group. (C) Representative photographs of TTC-stained heart sections obtained from I/R and SP+I/R groups at 7 days after inducing I/R. The percentages of infarct area to LV volumes are shown in the bar graph. **P<0.05* vs. corresponding controls using Student’s *t*-test. (D and E) Graphs of qRT-PCR analyses of targeted gene expression in the RA, LA, RV, LV, and apex of I/R and SP+I/R at 1 day. **P*<0.05 versus corresponding control using Student’s *t*-test.

**
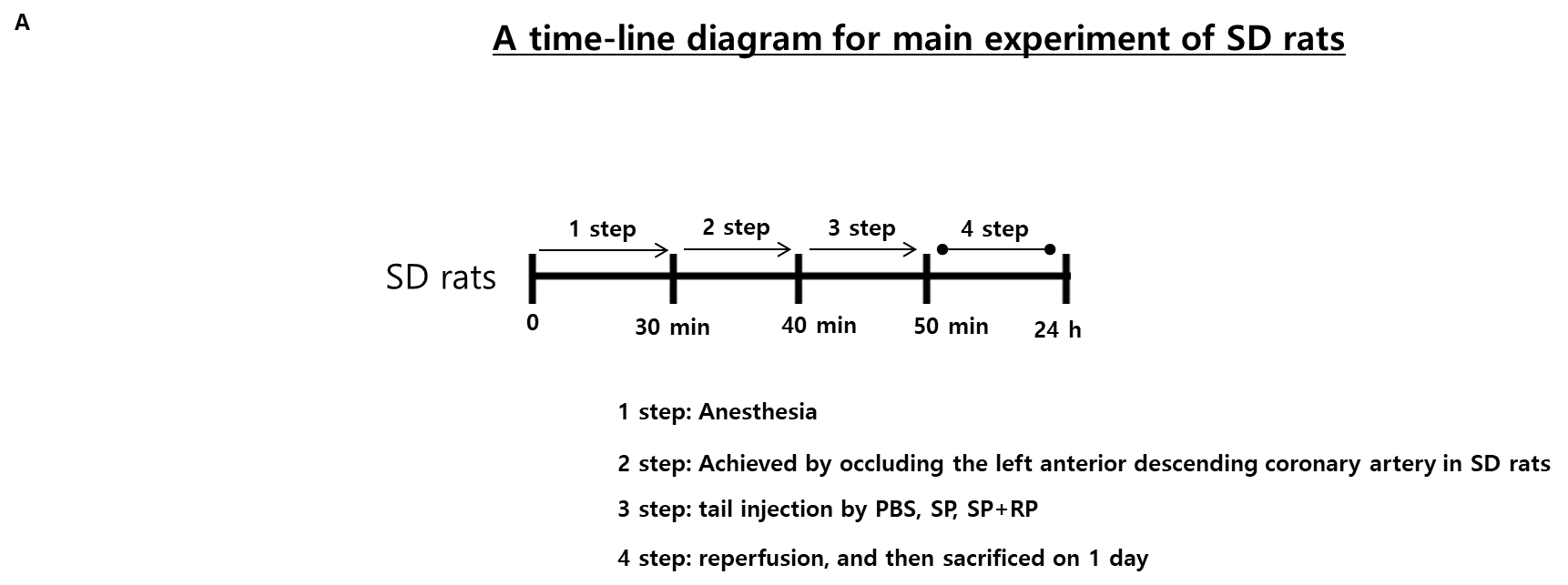
**

**
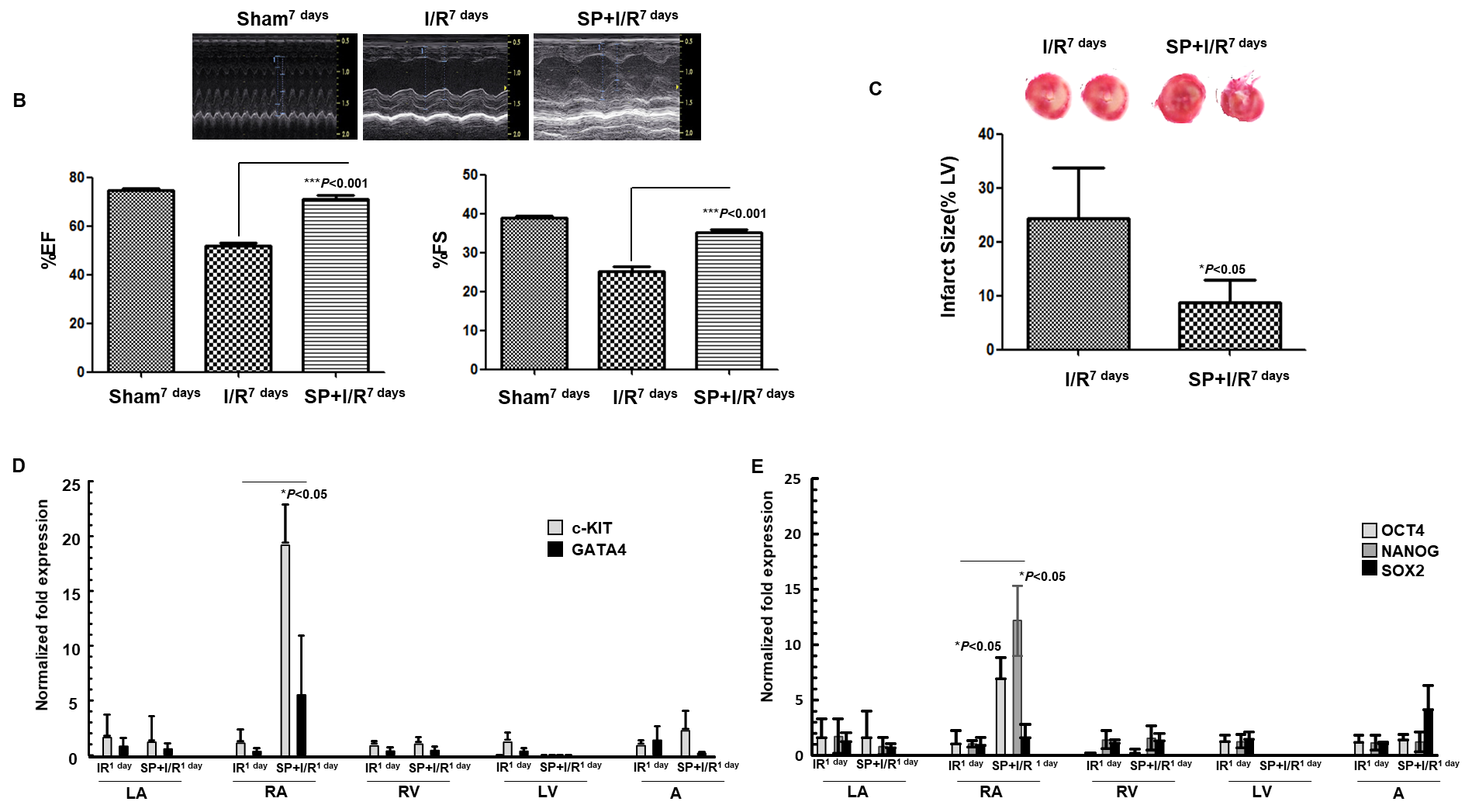
**

**Figure S2. RA^1 day I/R^ EDCs and RA^1 day SP+I/R^ EDCs did not show cTnl expression.** Confocal images of c-TnI (green) in the RA^1 day I/R^ EDCs and RA^1 day SP+I/R^ EDCs. Scale bars 20 μm.


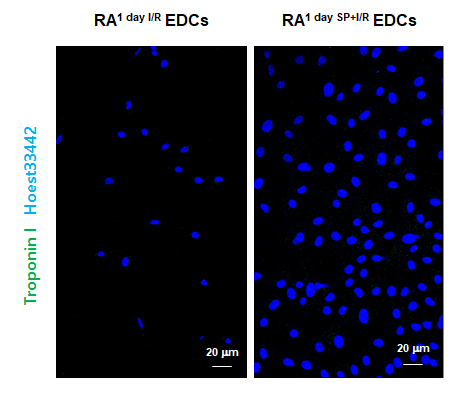


**Figure S3.** **Characterization of NK_1_R-expressing c-Kit^+^ CPCs.** (A) RT-PCR of purified c-Kit^+^ CPCs (1) and RA^I/R^ EDCs (2) for calculating the expression of markers. (B) Images of morphology of cardiosphere from purified c-Kit^+^ CPCs, BMSCs, and RA^I/R^ EDCs.


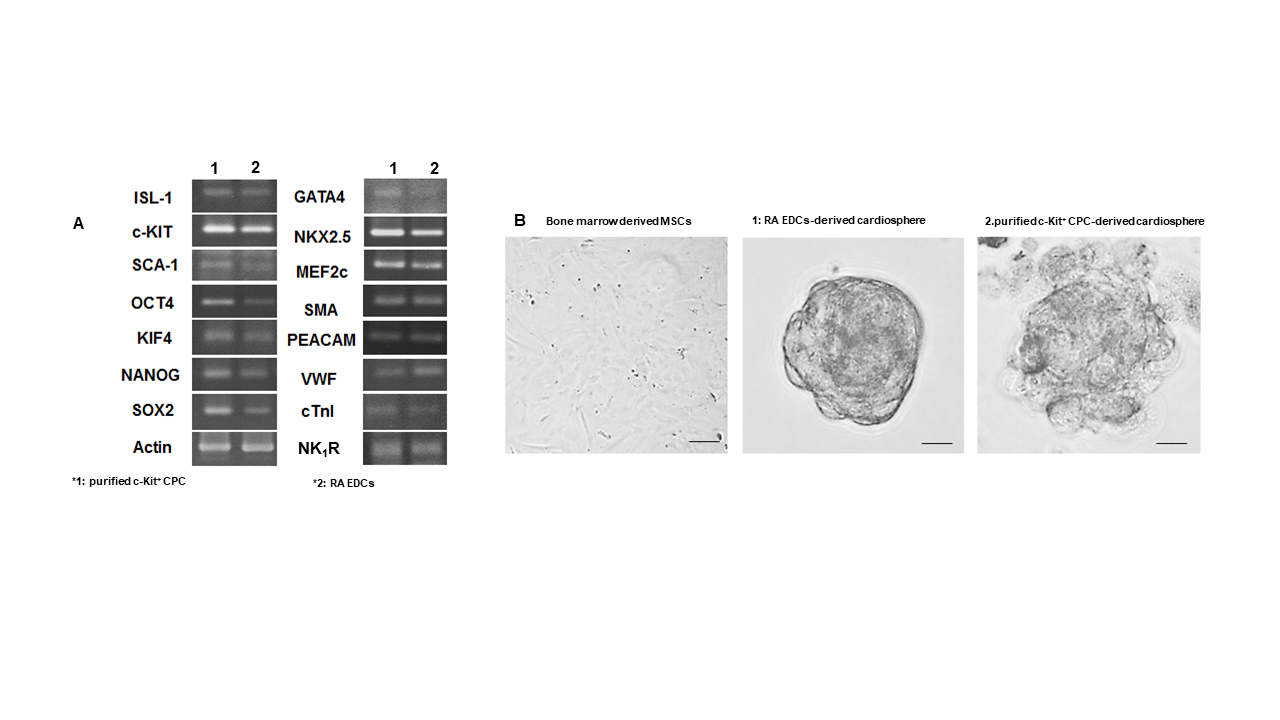


**Figure S4. NK_1_R inhibitor blocks SP-stimulated the cell proliferation, migration, and cardiosphere formation of RA c-Kit^+^ CPCs.** (A) c-Kit^+^ CPC proliferation with SP or SP+RP or neither was measured using the EZ-Cytox cell viability assay kit. ****P*<0.001, versus control using a one-way analysis of variance (ANOVA) followed by Tukey’s *post hoc tests*. (B) Graph indicating the rate of cell migration. *** *P<0.001*, versus control using a one-way analysis of variance (ANOVA) followed by Tukey’s *post hoc tests*. (C) Images of morphology of cardiosphere from purified c-Kit^+^ CPCs with SP or SP+RP or neither. Graphs showing the number of cardiospheres **P*<0.001, versus control using a one-way analysis of variance (ANOVA) followed by Tukey’s *post hoc tests*.


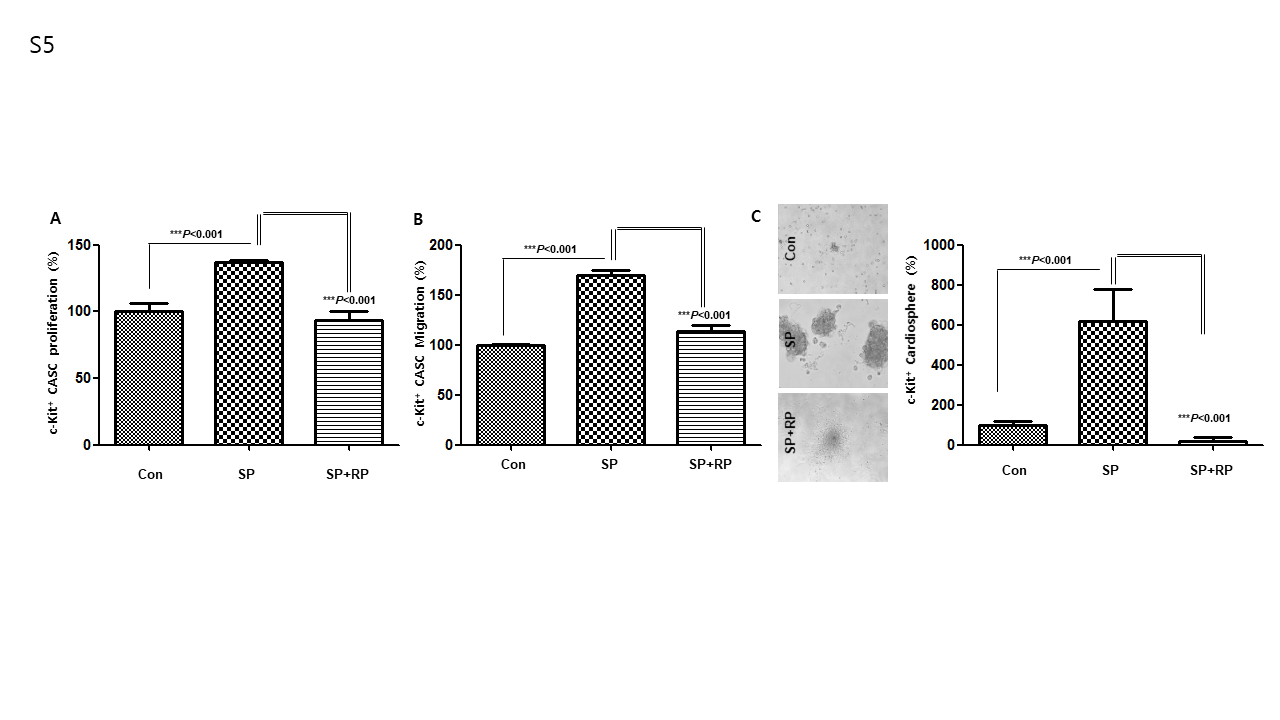


**Figure S5. NK_1_R inhibitor blocks SP-stimulated the cardiomyocyte differentiation of RA c-Kit^+^ CPCs.** Confocal images of α-actinin-labeled cardiomyocytes (red) and c-TnI (green) with SP or SP+RP or with neither for detecting co-expression of differentiation markers. Scale bars 20 μm.


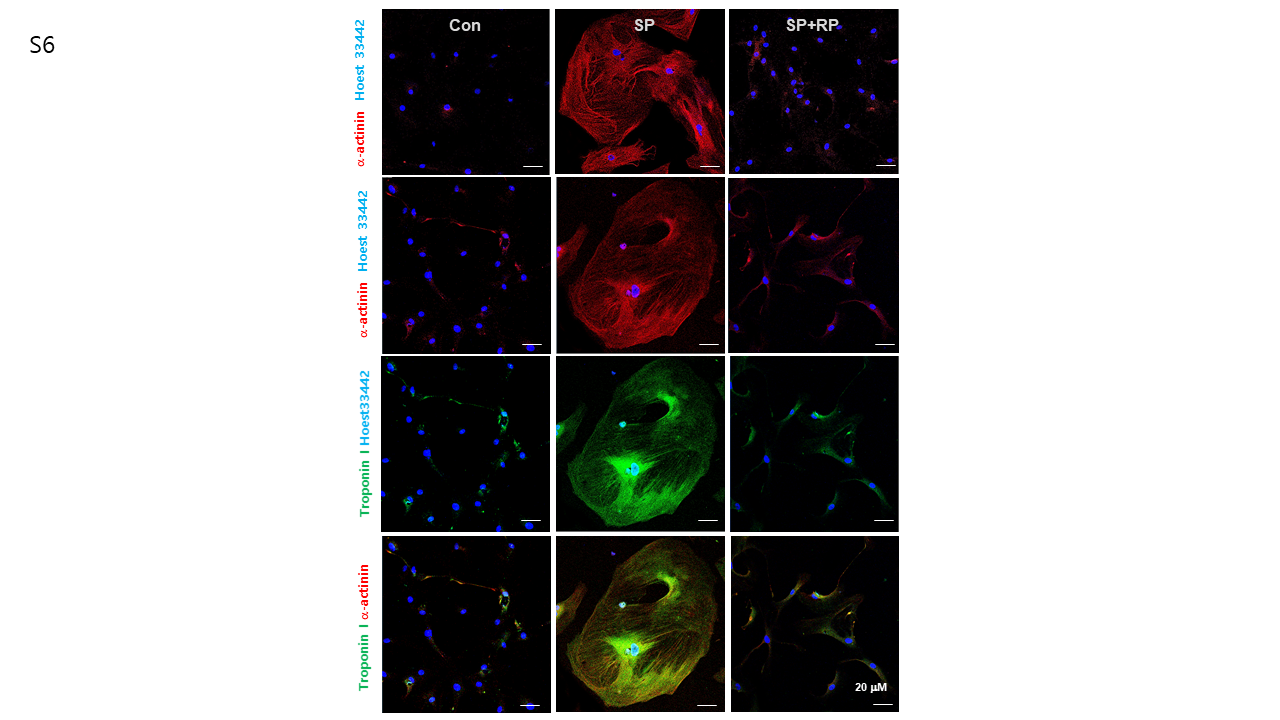

Supplement: Supplementary file 1 — Additional file 1: Fig. S1. The effects of SP on I/R-injured heart. Fig. S2. RA1 day I/R EDCs and RA1 day SP + I/R EDCs did not show cTnl expression. Fig. S3. Characterization of NK1R-expressing c-Kit+ CPCs. Fig. S4. NK1R inhibitor blocks SP-stimulated the cell proliferation, migration, and cardiosphere formation of RA c-Kit+ CPCs. Fig. S5. NK1R inhibitor blocks SP-stimulated the cardiomyocyte differentiation of RA c-Kit+ CPCs. [file 12860_2020_286_MOESM1_ESM.doc]
